# ﻿Description of one new species of the genus *Orthozona* Hampson, 1895 (Lepidoptera, Erebidae, Herminiinae) from China

**DOI:** 10.3897/zookeys.1153.79856

**Published:** 2023-03-13

**Authors:** Ting-Ting Zhao, Xin-Yu Zhang, Hui-Lin Han

**Affiliations:** 1 School of Forestry, Northeast Forestry University, Harbin, 150040, China Northeast Forestry University Harbin China; 2 Simianshan Forest Resource Service Center, Jiangjin District, Chongqing, 402296, China Simianshan Forest Resource Service Center Chongqing China; 3 Key Laboratory of Sustainable Forest Ecosystem Management-Ministry of Education, Northeast Forestry University, Harbin, 150040, China Northeast Forestry University Harbin China; 4 Northeast Asia Biodiversity Research Center, Northeast Forestry University, Harbin, 150040, China Simianshan Forest Resource Service Center Chongqing China

**Keywords:** Herminine moths, new species, southwest China, taxonomy

## Abstract

A new species of the genus *Orthozona* Hampson, 1895, *O.parallelilineata***sp. nov.**, is described from China. The new species is illustrated with images of adults and genitalia, and it is compared to similar species, *O.quadrilineata* and *Paracolaxcurvilineata*. A distribution map of this new species is also presented.

## ﻿Introduction

The genus *Orthozona* was erected by Hampson (1895) with *Madopaquadrilineata* Moore, 1882 as the type species from Darjiling, India. The genus has included as many as five species, namely *Orthozonabilineata* Wileman, 1915; *O.curvilineata* Wileman, 1915; *O.quadrilineata* (Moore, 1882); *O.karapina* Strand, 1920, and *O.rufilineata* (Hampson, 1895) (Poole 1989; Beccaloni et al. 2003). However, *O.bilineata* and *O.curvilineata* were transferred to *Paracolax* Hübner, [1825] by Owada (1992a) and Owada et al. (2021). To date, two species are known to occur in China: *O.karapina* and *O.quadrilineata* (Owada 1992b; Chen 1999; Wu et al. 2013). Smetacek and Kitching (2012) recorded a male specimen of *O.quadrilineata* from northwestern India and illustrated a female syntype of *O.quadrilineata* from Darjeeling, but they did not show the male and female genitalia. In the present study, a new species is described from Xizang Autonomous Region, China. Further study is needed to the understand the relatedness of *Orthozona* with *Paracolax*.

## ﻿Materials and methods

Specimens were collected in Xizang Autonomous Region, China, using a 220 V/450 W mercury lamp and a DC black light. Standard methods for dissection of the genitalia and preparation of the slide mounts were used (Kononenko and Han 2007). Photographs of the adults were taken with a Nikon D700 camera; genitalia slides were photographed with an Olympus photo-microscope, composited with Helicon Focus, and further processed with Adobe Photoshop CS6. The types of the two new species are deposited in the collection of Northeast Forestry University, Harbin, China.

Abbreviations for institutional collections are as follows:

**NEFU** Northeast Forestry University (Harbin, China);

**NSMT**National Museum of Nature and Science (Tsukuba, Japan).

## ﻿Taxonomic account

### ﻿Genus Orthozona

Taxon classificationAnimaliaErebidae

Hampson, 1895

9624E87A-B2BB-5B90-902B-595D05A4A58F

Orthozona Hampson, 1895, 94. Type species: Madopaquadrilineata Moore, 1882.

#### Diagnosis.

In morphology, both *Orthozona* and *Paracolax* Hübner, [1825] share some charactersas follows: the color of the forewings is ochre to dark ochre, the antemedial and postmedial line are distinct; the distal part of valva are tongue-like, the saccular process are developed; and the ductus bursae have a pair of sclerotized stripes. However, these genera can be distinguished by the following features: the antemedial and postmedial lines in *Orthozona* are inwardly oblique or slightly curved but always paralleled with each other, whereas the two lines in *Paracolax* are oblique or wavy and seldom parallel; the reniform spots in *Orthozona* are not obvious, while these spots in *Paracolax* are obvious; the saccular process of *Orthozona* is finger-shaped, slightly separated from the valva, and slightly sclerotized, whereas the saccular process in *Paracolax* is variously shaped, e.g. digitiform, conical, truncate, etc.; and the corpus bursae of *Orthozona* is sac-like, with a single signum, while the corpus bursae of *Paracolax* is very long, more than twice as long as the ductus bursae, and with or without signum (Owada 1992a; Chen 1999; Smetacek and Kitching 2012; Wu 2014).

#### Description.

The proboscis is developed; the labial palpus in males is sickle-shaped, mostly covered with scale tufts ventrally; labial palpus in females with the 2^nd^ segments straight and the 3^rd^ segments upturned; the antenna is filiform. Thorax: quite stout; the forewing is broad, with the outer margin broad and slightly excurved; in many species of this genus, the medial line of the hindwing is indistinct and the subterminal line is slightly arched. Abdomen: slender, slightly lighter than the thorax; the uncus is slender, with a hooked apex; the tegumen is narrow and triangular; the saccus is U-shaped; the valva is simple and weakly sclerotized, with saccular process; the vesica is covered minutely granular and bears a basal cornutus; in females, the analis papili is short, the apophyses posteriores and anteriores are moderate in length, the ductus bursae is short, the corpus bursae have more extensive microspines, and a signum is present.

### ﻿ Orthozona parallelilineata

Taxon classificationAnimaliaErebidae

Zhao, Zhang & Han sp. nov.

85A357A7-B112-5CE4-B40A-E09D2A6381B9

https://zoobank.org/5DE18C80-120E-445A-A501-54900F5F7D91

**Figures 1–6.**
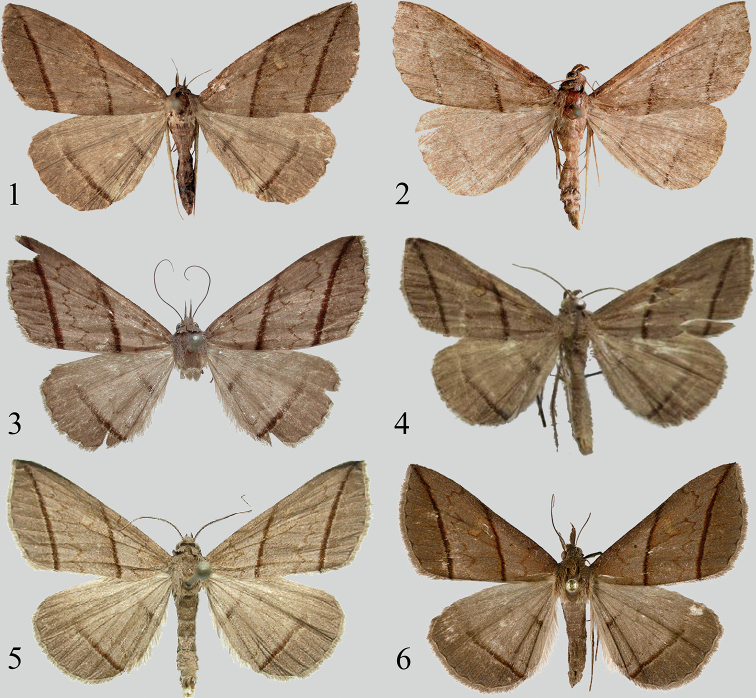
*Orthozona* and *Paracolax* spp., adults **1***O.parallelilineata* sp. nov., female, holotype, Xizang **2***O.parallelilineata* sp. nov., male, paratype, Xizang **3***O.quadrilineata* (Moore, 1882), female, Sikkim (from Dr Mamoru Owada) **4***O.quadrilineata* (after Smetacek and Kitching 2012) **5***P.curvilineata* (Wileman, 1915), male, Taiwan (from Dr Shipher Wu) **6***P.curvilineata*, female, Taiwan (from Dr Shipher Wu).

**Figures 7–11.**
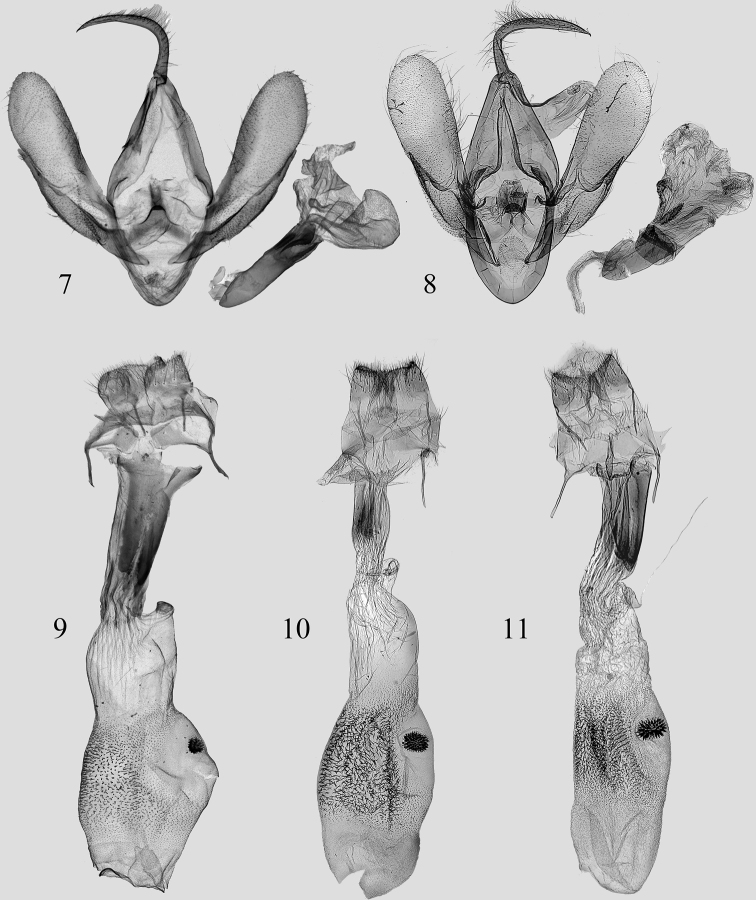
Genitalia of *Orthozona* and *Paracolax* spp. **7***O.parallelilineata* sp. nov., paratype, genit. prep. zxy-0103-1 **8***P.curvilineata* (Wileman, 1915), Taiwan (from Dr Mamoru Owada, genit. prep. NSMT4187, in NSMT) **9***O.parallelilineata* sp. nov., holotype, genit. prep. zxy-0132-2 **10***O.quadrilineata* (Moore, 1882), Sikkim (from Dr Mamoru Owada, genit. prep. NSMT4373, in NSMT) **11***P.curvilineata* (from Dr Mamoru Owada).

**Figures 12–14.**
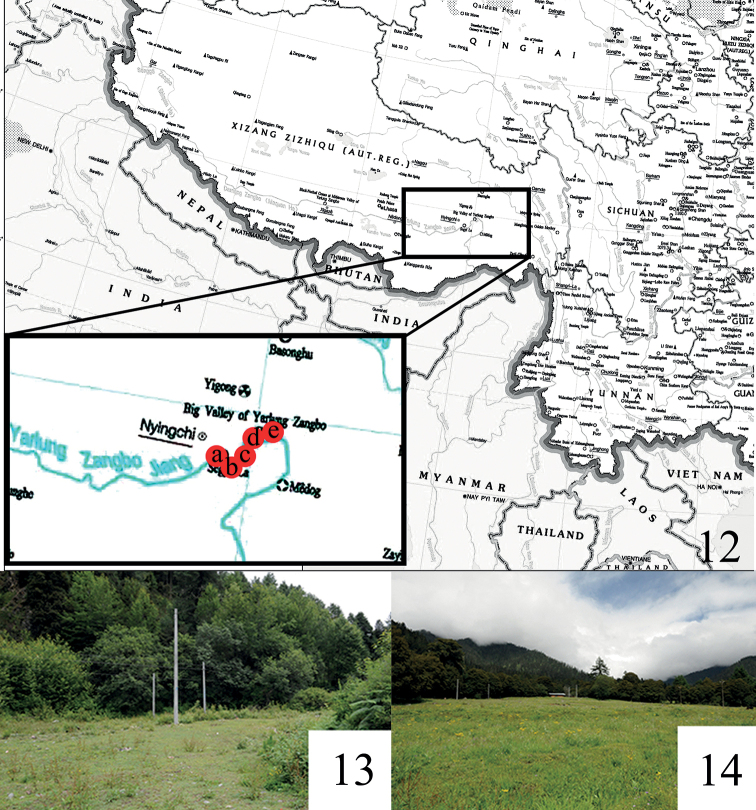
Distribution and collecting sites of *Orthozona* spp. **12** distribution map: a = Mount Sejila; b = Nadengzuo Village; c = Lulang Town; d = Layue Village; e = Pailong Countryside **13** collection site at Nadengzuo Village, Linzhi City, Xizang Autonomous Region **14** collection site in Luluang countryside, Linzhi City, Xizang Autonomous Region.

Figs 1 , 2 , 7 , 9 , 12–14

#### Material examined.

***Holotype***: ♀, China; Xizang Autonomous Region, Linzhi City, Lulang Town; 19.VIII.2014; H.L. Han leg.; genitalia No. zxy-0132-2; coll. NEFU. ***Paratypes***: 1 ♀, China; Xizang Autonomous Region, Linzhi City, Pailong Countryside; 22–23.IX.2011; H.L. Han leg.; genitalia No. ztt-5280-2; 1 ♀ • Xizang Autonomous Region, Linzhi City, Nadengzuo Village; 14–15.VIII.2014; H.L. Han leg.; genitalia No. ztt-5286-2; 3 ♂♂, 2 ♀♀ • Xizang Autonomous Region, Linzhi City, Nadengzuo Village; 17.VIII.2014; H.L. Han leg.; genitalia No. zxy-0099-2, zxy-0103-1, ztt-5278-2, ztt-5281-1, ztt-5284-1; 3 ♀♀, 1 ♂ • Xizang Autonomous Region, Linzhi City, Mount Sejila; 20.VIII.2014; H.L. Han leg.; genitalia No. hhl-5279-1, hhl-5282-2, hhl-5283-2, hhl-5291-2; 1 ♀ • Xizang Autonomous Region, Linzhi City, Mount Sejila; 22.IX.2016; Z.H. Pan leg.; genitalia No. hhl-5289-2; 3 ♂♂ • Xizang Autonomous Region, Linzhi City, 13.VIII.2017; H.L. Han leg.; genitalia No. hhl-5285-1, hhl-5288-1, hhl-5293-1; coll. NEFU.

#### Diagnosis.

*O.parallelilineata* sp. nov. (Figs 1, 2) is superficially similar to *O.quadrilineata* (Figs 3, 4) and *P.curvilineata* (Figs 5, 6) but can be separated from these species by the following characters. In the male genitalia (Fig. 7), the valva is narrower (in *P.curvilineata*, the cucullus is roundish and broader); the sacculus process is approximately 3/4 the length of valva, narrow, and the distal part is thin, finger-shaped (in *P.curvilineata*, the length of sacculus processes is obviously less than 1/2 of the valva, and the sclerotized is apically pointed); the saccus is narrower (in *P.curvilineata*, the saccus is broader); the phalli are slightly longer (in *P.curvilineata*, the phalli are shorter and stouter).

The characters of female genitalia (Fig. 9) are very similar to those of *O.quadrilineata* (Fig. 10) and *P.curvilineata* (Fig. 11); the corpus bursae is covered with sparse spinules (in *O.quadrilineata* and *P.curvilineata*, the spinules are more dense); the ductus bursae has two long, sclerotized longitudinal bands, which is similar to that of *P.curvilineata* (in *O.quadrilineata*, the ductus bursae has a small sclerotized band).

#### Description.

**Adult** (Figs 1, 2). Wingspan 37–39 mm in both sexes. Head: dark brown; labial palpus sickle-shaped in male, extended forward in female; antenna filiform. Thorax: patagium and tegula dark brown; mesothorax and metathorax light ochre-grey. Forewing light ochre-brown, diffused with brown scales; antemedial line dark brown, excurved, with a deep, excurved dent in Cu_2_–1A+2A area; medial line inwardly oblique, dark blackish brown, broadly band-shaped, slightly curved inwards; postmedial line narrow, brown, waved on the vein, with a large, excurved dent at the costal margin region, and a large, incurved dent in Cu_2_–1A+2A area; subterminal line inwardly oblique, dark blackish brown, broadly band-shaped, slightly curved inwards, parallel to the medial line, and running from the apex to inner margin close to the tornus; terminal line grey, narrow; orbicular spot small, brown, distinct, encircled with a halo; reniform spot oval; a flap with a fringe of scales underlapping the forewing costa below over ca 2/3 of its length in male. Hindwing slightly lighter than forewing; media line brown, more prominent in inner margin region; postmedial line very narrow, almost not visible; subterminal line broad, blackish brown to brown, gradually noticeable from costal to inner margin; discal spot obscure. Both forewing and hindwing with a discal spot on the ventral side, respectively. Abdomen: light ochre-grey, rather slender, terminus with yellow tufts.

***Male genitalia*** (Fig. 7). Tegumen broad, slightly longer than vinculum. Vinculum narrow, U-shaped. Saccus with a rounded and rough central plate. Valva narrow, insole-shaped, gradually widening from base to cucullus; sacculus moderately sclerotized, narrow, band-shaped, ca 3/4 length of valva; sacculus process sclerotized, narrow, finger-shaped, weakly split from the valva; costa flattened, tapering from basal to terminal part, ca 2/3 length of valva; cucullus smoothly arched. Uncus long and slender, tapering from basal part to top, curved at 1/3 from base, apically sharp. Juxta inverted V-shaped, slightly concavea on both sides, papillate and sclerotized apically. Phallus straight; coecum narrow, ca 1/3 length of aedeagus; carina with a short and narrow spine, and with a sclerotized and short band ventrally. Vesica has four membranous diverticula, densely covered with small grains; basal part of vesica with a reversed, curved, saw-toothed cornutus; subbasal diverticulum is the largest.

***Female genitalia*** (Fig. 9). Ostium bursae broad and straight. Dorsal side of 8^th^ segment with a pair of subcircular, slightly sclerotized areas, connected to base of apophysis anterior. Ductus bursae flattened, shorter than 1/2 length of corpus bursae, with two long, sclerotized longitudinal bands ca 2/3 length of ductus bursae. Corpus bursae membranous; anterior part densely covered with small granulations, with an oval signum covered with small, stout spines; posterior half smooth, covered with longitudinal wrinkles. Apophysis anterior thicker and longer than apophysis posterior. Papilla analis thick and short.

#### Distribution.

China (Xizang: Linzhi) (Fig. 12). This species occurs in grasslands around coniferous and broad-leaved mixed forests in the Xizang Autonomous Region (Figs 13, 14). It flies in the dry season. Specimens were captured with UV light in August.

#### Etymology.

The species is named for the parallel medial and subterminal lines.

#### Remarks.

*Orthozonaquadrilineata* and *Paracolaxcurvilineata* are very similar in external appearance and genitalia in both sexes. However, they are not discussed further here due to the poor condition of their materials; the relationship between these three species deserves in-depth study when the materials become available. In this paper, one female of *O.quadrilineata* was collected by Dr M. Owada on 25 September 1983 in Sikkim, India (genitalia slide no. NSMT4373).

## Supplementary Material

XML Treatment for Orthozona

XML Treatment for Orthozona parallelilineata
